# Biology, detection, and clinical implications of circulating tumor cells

**DOI:** 10.15252/emmm.201303698

**Published:** 2014-11-14

**Authors:** Simon A Joosse, Tobias M Gorges, Klaus Pantel

**Affiliations:** Department of Tumor Biology, Center of Experimental Medicine, University Medical Center Hamburg-EppendorfHamburg, Germany

**Keywords:** Disseminating tumor cells (DTC), EMT, metastasis, tumor cell dormancy, tumor cell plasticity

## Abstract

Cancer metastasis is the main cause of cancer-related death, and dissemination of tumor cells through the blood circulation is an important intermediate step that also exemplifies the switch from localized to systemic disease. Early detection and characterization of circulating tumor cells (CTCs) is therefore important as a general strategy to monitor and prevent the development of overt metastatic disease. Furthermore, sequential analysis of CTCs can provide clinically relevant information on the effectiveness and progression of systemic therapies (e.g., chemo-, hormonal, or targeted therapies with antibodies or small inhibitors). Although many advances have been made regarding the detection and molecular characterization of CTCs, several challenges still exist that limit the current use of this important diagnostic approach. In this review, we discuss the biology of tumor cell dissemination, technical advances, as well as the challenges and potential clinical implications of CTC detection and characterization.

## Biology of circulating tumor cells

### Introduction

**Figure 1:**
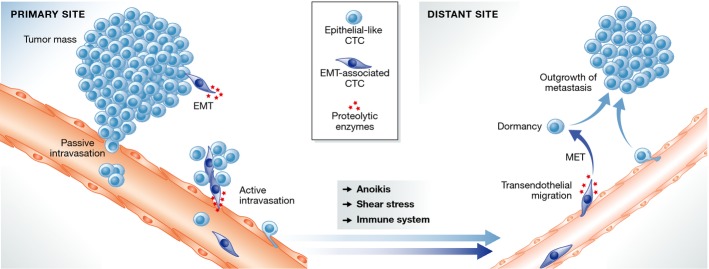
Metastatic cascade Tumor cells may enter the bloodstream passively or actively via biological events such as EMT or centrosomes amplification. Disseminating tumor cells must overcome several hurdles including anoikis, shear stress in the bloodstream, and the immune system in-and outside of the blood circulation. Once at a distant site, tumor cells may extravasate, undergo MET, and grow locally to become a metastasis or remain in dormancy.

The ability of a malignant tumor to become metastatic begins with the hallmarks of motility and invasiveness (Hanahan & Weinberg, 2011). When individual cells or clusters of cancer cells acquire the ability to separate and move away from the primary tumor mass, migrate through the surrounding tissue, and enter the lymphatic system and/or blood circulation, the metastatic trait of a given tumor becomes manifest (Fig1). Tumor cells that escape their primary microenvironment need to escape anoikis and might be challenged by the host's immunologic defenses. Although the rate of tumor cell release in cancer patients is unknown, experimental models indicate that millions of tumor cells are continuously dispersed through the body, although only few of these cells might reach a distant organ, survive in a dormant state, evade the immune system and systemic therapy, and eventually grow into an overt metastasis (Chambers *et al*, 2002; Kang & Pantel, 2013). Adaption to a new microenvironment and proliferation of a single tumor cell in a distant site requires special traits (plasticity) that the cell must already have or acquire in order to develop to an overt metastasis detectable by clinical procedures. Tumor cells may stop cell division processes after dissemination but may persist in a quiescent status until environmental conditions provide appropriate signals to start proliferation again. This quiescent state, for which the underlying mechanisms are largely unknown, is called clinical cancer dormancy (Uhr & Pantel, 2011; Kang & Pantel, 2013) and may last more than 10 years. There is now increasing evidence that the microenvironment plays a critical role in this process (e.g., osteoclasts as regulators of osteolytic bone metastases (Lu *et al*, 2011; Ell *et al*, 2013)), in addition to changes in the tumor cells themselves (e.g., acquisition of new mutations).

### CTC migration: mobility and motility

Cancer metastasis has been correlated with specific genetic biomarkers such as mutations, chromosomal aberrations, and gene expression patterns (Nguyen & Massague, 2007; Wrage *et al*, 2009; Wikman *et al*, 2012; Hohensee *et al*, 2013). Nevertheless, whether CTCs enter the blood circulation through an active migration process, by passive means, or both, remains clinically and scientifically an unresolved question (Joosse & Pantel, 2013). In this review, we employ the terms *motile* and *mobile cells* to describe the differences between active and passive migration of the tumor cells.

*Motile* cancer cells are cells that are able to move on their own accord and that has thus gained the ability to move through the extracellular matrix and penetrate basement membranes and endothelial walls upon intravasation and extravasation. These active migration mechanisms imply modification of cell morphology, position, and surrounding tissue (Friedl & Alexander, 2011). Furthermore, cancer cells may infiltrate as single entities, in clusters, in strands, or in single (Indian) files as observed in lobular breast carcinoma. Single cells must weaken or completely lose their adhesive bonds with neighboring tumor cells for infiltration, whereas collective migration requires stable cell–cell adhesion and multicellular coordinated movement (Friedl & Gilmour, 2009). These clusters frequently comprise of different cell morphologies, that is, both epithelial-and mesenchymal-like. Collective migration may require a leader cell with mesenchymal features, able to create a path for the trailing tumor cells through the surrounding tissue (Friedl & Wolf, 2009).

*Mobile* cancer cells are moved by external forces such as growth of the tumor, mechanical forces, or friction which cause them to be dragged or pushed out of place (Camara *et al*, 2006; Fornvik *et al*, 2013). Although a vast amount of literature is available about the processes involved in active migration of cancer cells through the extracellular matrix, less is known concerning the passive dissemination of cancer cells and how they may be forced into the blood circulation. Carcinomas induce the formation of new blood vessels by the secretion of the vascular endothelial growth factor (VEGF) to facilitate the supply of nutrients and oxygen for growth. This process called angiogenesis, often results in leaky vessels caused by weak interconnections of the vessel's endothelial cells and intercellular openings (McDonald & Baluk, 2002). Due to outward pushing of the tumor during growth, single or clusters of tumor cells can be forced through the leaky vessels, thus ending up in the blood circulation as ‘accidental CTCs’. Furthermore, tumor cells may be passively moved through the micro-tracks created by other tumor cells by proteolysis or through other pre-existing tissue structures (Friedl & Wolf, 2009).

Because the ‘accidental CTCs’ from epithelial malignancies are more likely to have retained their original phenotype, detection with epithelial-specific markers such as the epithelial cell adhesion molecule (EpCAM) would be feasible. On the other hand, tumor cells that have transformed to a more mesenchymal-like phenotype, and thus exhibit greater plasticity, may prove to be less easily detected by conventional EpCAM-based detection methods (Brabletz, 2012; Joosse & Pantel, 2013). Nevertheless, new evidence shows that a phenotypical transformation is not always required for tumor cell motility (Aceto *et al*, 2014; Godinho *et al*, 2014).

### Epithelial–mesenchymal plasticity

Epithelial cells adhere to their neighboring cells through, among others, adherens junctions via binding of cadherins. Carcinoma cells are of epithelial origin and experience the same cell–cell interaction through adhesion molecules such as cadherins, claudins, or plakoglobin; these interactions prevent them from spreading in the first place (Pantel *et al*, 1998).

Normal epithelial cells show remarkable plasticity as they are able to reprogram and undergo dynamic and reversible transitions between the epithelial and mesenchymal cell phenotype (Tam & Weinberg, 2013). This de-differentiation process known as epithelial-to-mesenchymal transition (EMT) occurs frequently throughout embryogenesis because cells continuously migrate to form tissues and organs. EMT also plays an important role in wound healing and tissue regeneration (Nieto, 2013). Not surprisingly, carcinoma cells also make use of EMT as they become invasive. Indeed, they typically feature loss of cell–cell adherence proteins such as cadherin, followed by loss of apico-basal polarity, and finally, gaining the ability to migrate and invade (Thiery, 2002). EMT can be triggered by paracrine signaling of TGF-beta, WNT, platelet-derived growth factors, or interleukin-6 (IL-6) but can also be induced by nicotine, alcohol, and ultraviolet light (Thiery *et al*, 2009; Tam & Weinberg, 2013; Kishi *et al*, 2014). These triggers activate transcription factors such as Snail, Twist, and Zeb that maintain the mesenchymal phenotype by autocrine signaling (Tam & Weinberg, 2013). Because of the loss of tight and adherens junctions, as well as cytoskeletal changes, typical epithelial markers such as EpCAM and E-cadherin are down-regulated, keratin expression is altered, and finally, up-regulation of mesenchymal markers such as vimentin is observed during EMT (Joosse *et al*, 2012). Consequently, mesenchymal-like CTC subpopulations are difficult to identify in the hematopoietic cell environment which is also of mesenchymal origin (Joosse & Pantel, 2013). Of note, single tumor cells found in the blood of breast cancer patients exhibit EMT-associated changes, while cell clusters appear to require a partial EMT so that these cells possess the migratory abilities of a mesenchymal cell but retain the cell–cell interaction profile of an epithelial cell (Yu *et al*, 2013).

### Non-EMT-associated motility

Centrosome amplification has also been linked to de-differentiation and recently been shown to induce invasion in cancer (Ghadimi *et al*, 2000; Lingle *et al*, 2002; Godinho *et al*, 2014). The centrosomes form the main microtubule organizing center in mammalian cells and facilitate chromosomal separation during mitosis via the meiotic spindle apparatus but also participate in the organization of flagella and cilia. EMT is thought not to play a role in the invasion of tumors with centrosome amplification since expression of E-cadherin is retained. Cell–cell adhesion appears instead reduced downstream of Rac1 signaling by increased Arp2/3-dependent actin polymerization (Godinho *et al*, 2014). Another form of non-EMT-associated dissemination, which is thought to substantially contribute to metastasis, is the release of circulating tumor cell clusters (Aceto *et al*, 2014). These clusters are constituted of 2–50 tumor cells held together by plakoglobin-dependent intercellular adhesion. Clustered cells are less likely to undergo anoikis and have an increased likelihood of being trapped in narrow blood vessels, thus favoring extravasation into distant organs.

Therefore, since not all metastatic tumor cells seem to lose their epithelial-like characteristics by EMT, it is important to consider CTCs with epithelial properties too in order to detect the metastasis-initiating cells (MICs).

### CTCs in circulation

Once in the bloodstream, CTCs face several natural obstacles that hinder the metastatic process. First are the enormous shearing forces and collisions with blood cells, generated by blood flow. Although shear stresses decrease the number of viable cancer cells dramatically, tumor cells that underwent EMT seem to be more resistant against these forces compared to epithelial tumor cells (Mitchell & King, 2013). Second, CTCs must survive in the bloodstream without their cell–matrix interactions, an occurrence that would normally trigger apoptosis through a process called anoikis. Resistance against anoikis, however, is made possible in CTCs by activated tropomyosin-related kinase B (TrkB) that suppresses caspase-associated apoptosis and enables the cells to survive in liquid suspension (Douma *et al*, 2004). The third obstacle that CTCs face in the blood is the activity of the immune system. In colorectal cancer, immune escape is obtained by up-regulation of CD47 that prevents CTCs from macrophage and dendritic cell attack (Steinert *et al*, 2014). Finally, cancer cells must eventually leave the blood circulation, which requires binding to the endothelium lining the vessels. Because platelets enhance this binding, inhibition of platelet aggregation by for instance aspirin can decrease stable tumor cell binding to activated platelets (Uppal *et al*, 2014).

### Tumor cell homing and dormancy

Extravasation starts when CTCs slow down in small capillaries, attach to the endothelium, and finally undergo transendothelial migration (Reymond *et al*, 2013). One of the most frequent CTCs homing sites are the bones, including for primary malignancies such as colorectal and lung cancer that less typically metastasize to the bone (Pantel & Brakenhoff, 2004; Braun *et al*, 2005; Riethdorf *et al*, 2008). It is therefore thought that the bone marrow functions as a reservoir for disseminated tumor cells (DTCs) (Wikman *et al*, 2008; Kang & Pantel, 2013). In breast cancer, it may take many years up to decades after surgery of the primary tumor until metastases become evident (Goss & Chambers, 2010; Uhr & Pantel, 2011). During this time, bone marrow DTCs (Janni *et al*, 2011) as well as CTCs derived from the DTCs (Meng *et al*, 2004) may be found in these patients. DTCs in bone marrow may linger in a dormant state, thus evading systemic therapy while waiting for the appropriate trigger to resume proliferation (Bragado *et al*, 2013; El Touny *et al*, 2014). The basis for tumor cell dormancy may be the initial EMT process itself. In squamous cell carcinoma, spatiotemporal regulation of the epithelial–mesenchymal transition is essential for the dissemination and eventual metastasis (Tsai *et al*, 2012). The mesenchymal phenotype of CTCs that underwent EMT promotes motility but does not favor growth (Celia-Terrassa *et al*, 2012). Indeed, cancer cells must undergo a reverse mesenchymal-to-epithelial transition (MET) to acquire the ability to proliferate and thus form a metastatic tumor. It has therefore been suggested that tumor cells with an intermediate phenotype can most efficiently disseminate and grow at the distant sites (Bednarz-Knoll & Weinberg, 2012; Tam & Weinberg, 2013).

## Enrichment and detection of CTCs

### Enrichment of CTCs

**Figure 2:**
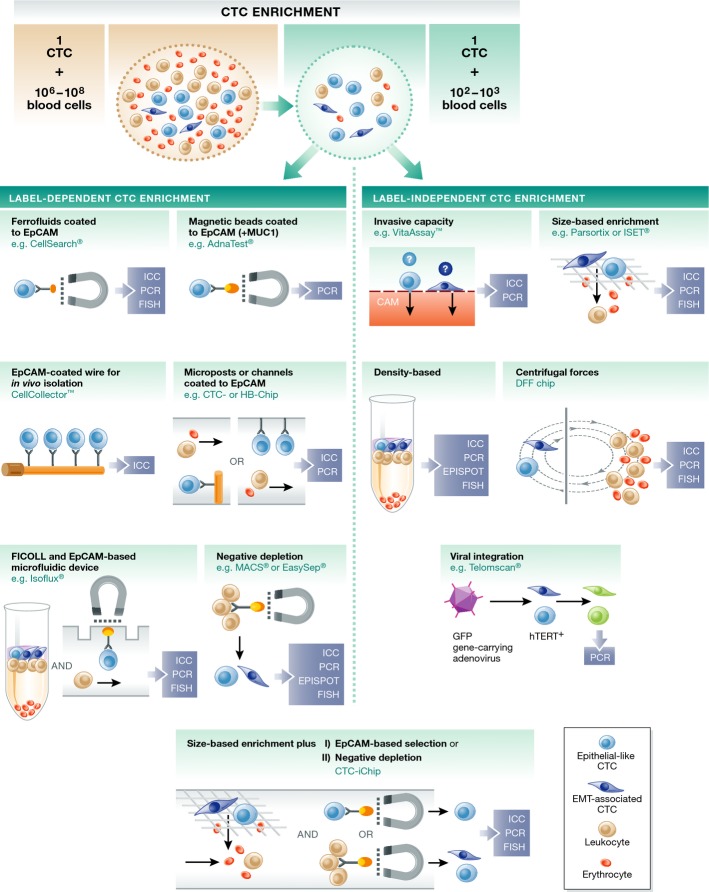
CTC enrichment and identification Enrichment strategies for CTCs can be separated into label-dependent and label-independent techniques. Among label-dependent techniques, immunomagnetic-based assays targeting the EpCAM protein are the most commonly applied. Label-independent enrichment methods include size-based or density-based approaches. Additionally, negative depletion or the invasive capacity of tumor cells can be used. A combination of different enrichment strategies is also practicable. Captured tumor cells are ready for molecular characterization by immunocytochemistry (ICC) using antibodies for tumor-specific markers or by PCR approaches targeting tumor-specific mRNA or DNA sequences. Another possibility is to detect viable cells by protein secretion (EPISPOT). Additionally, fluorescence in situ hybridization (FISH) can be used for the detection of tumor-specific gene aberrations.

CTCs are infrequent and appear at an estimated level of one against the background of millions (10^6^–10^7^) of surrounding normal peripheral mononuclear blood cells (PBMCs) (Alix-Panabieres *et al*, 2012). The occurrence rate might even be lower in cancer patients without obvious metastases (Rink *et al*, 2012; Rack *et al*, 2014). Hence, selective enrichment of tumor cells and/or systematic removal of PBMCs and red blood cells (RBCs) is required to detect CTCs in the blood of a cancer patient. To date, more than 40 techniques have been developed for CTC detection (Parkinson *et al*, 2012) and novel strategies are published continuously. CTC enrichment and detection methods have been classified based on whether they exploit the physical or biological properties of the cells (Alix-Panabieres & Pantel, 2014). However, as many enrichment strategies rely on positive selection (usually targeting EpCAM), CTC assays are more commonly grouped into label-dependent and label-independent approaches (Fig2). Among the numerous EpCAM-based CTC detection technologies, the semi-automated CellSearch ® system is the most frequently used system and at present the only one cleared by the U.S. FDA. Through this approach, CTC counts have been associated with an independent prognostic power on progression-free survival (PFS) and overall survival (OS) in primary and metastatic disease (Cristofanilli *et al*, 2004; de Bono *et al*, 2008; Cohen *et al*, 2009; Bidard *et al*, 2014; Rack *et al*, 2014). Due to its clinical relevance, high reproducibility, and FDA clearance, CellSearch ® can be considered as a benchmark for all other CTC detection methods appearing on the market. The AdnaTest ® is another epithelial marker-based research tool that positively enriches CTCs from a blood sample. Using this approach, the presence or disappearance of CTCs was shown to be a prognostic and predictive marker in a study on metastatic breast cancer (Tewes *et al*, 2009). Microfluidic device platforms like the CTC-or the Herringbone (HB)-Chip seem to be promising alternatives to selectively capture EpCAM-positive CTCs in cancer patients (Nagrath *et al*, 2007; Maheswaran *et al*, 2008). A combination of anti-EpCAM and anti-MUC1 capture in a single microfluidic device may further result in an improved capture performance of CTCs (Thege *et al*, 2014). Another chip-based platform combines a size-based filtration with an affinity-based enrichment strategy, thus enhancing the chance of systematic removal of PBMCs and RBCs (CTC-iChip) (Ozkumur *et al*, 2013). Fluxion Biosciences has lately introduced a commercially available microfluidic technology (IsoFlux ®) with magnetic isolation zones to isolate EpCAM-positive tumor cells from biological samples. Using the IsoFlux ®, recovery rates between 74 and 85% of EpCAM^low^-(MDA-MB-231) and EpCAM^high^ (SKBR3)-expressing tumor cells could be obtained (Harb *et al*, 2013). To circumvent sample volume limitations, GILUPI GmbH has designed an EpCAM-coated wire (CellCollector ™) to capture CTCs *in vivo* (Saucedo-Zeni *et al*, 2012). This device is positioned through a cannula into the vein of a cancer patient. It is estimated that during the 30-min application time, up to 1.5 l of blood flows over the detector, thus increasing the yield of detectable CTCs.

However, a major caveat is that metastasis formation might require EMT, which in turn might cause loss of CTC detection due to the down-regulation of EpCAM. EMT-related changes such as the down-regulation of EpCAM and Keratins or the up-regulation of EGFR and vimentin have already been described on CTCs and DTCs (Bartkowiak *et al*, 2009; Armstrong *et al*, 2011; Gorges *et al*, 2012; Joosse *et al*, 2012; Kasimir-Bauer *et al*, 2012; Gasch *et al*, 2013). Hence, EpCAM-based enrichment technologies should be re-worked for the comprehensive detection of epithelial (epithelial^+^/mesenchymal^−^), complete EMT (epithelial^−^/mesenchymal^+^), and intermediate EMT (epithelial^+^/mesenchymal^+^) CTC phenotypes.

Depletion of CD45-positive leukocytes (negative selection) is a preferred approach to capture CTCs lacking adequate expression of the EpCAM protein. Using this strategy, viable tumor cells have already been isolated, characterized, and cultured (Deneve *et al*, 2013). CD45 depletion can be combined with other label-independent methodologies (e.g., red blood cell lysis or density gradient centrifugation) to improve the yield and to displace unwanted erythrocytes (Liu *et al*, 2011). However, red blood cell lysis or density gradient centrifugation may lead to a loss of tumor cells resulting in false-negative results. The novel CTC-iChip can deplete hematopoietic cells combining this strategy with a size-based enrichment step (Ozkumur *et al*, 2013). In the antigen-independent mode, this system was able to isolate CTCs from several types of cancers that had lost or never had the epithelial marker characteristics (triple-negative breast cancer and melanoma). A combination of red blood cell lysis, depletion of CD45^+^ leukocytes, and size-based filtration has also been shown to be feasible in non-small cell lung, breast, colon, and esophageal cancer patients (Wu *et al*, 2014). Membranous filter devices like the ISET ® system or the ScreenCell ® approach appear to enrich CTCs with a high recovery rate as well. However, in particular, EMT-related CTCs may not be stiffer than leukocytes and CTCs are not always larger than leukocytes and might therefore not be retained by these filters. Moreover, even if tumor cells are captured on the membrane, they may be difficult to detach for further molecular examination. The Parsortix system is a novel separation device that captures viable tumor cells based on their physical properties. This system allows a size-based identification of cancer cells within the capture cassette device or harvesting of the enriched tumor cells outside the cassette. The harvested tumor cells are easily accessible and ready for downstream analysis, such as, next-generation sequencing (NGS), qPCR, FISH, ICC, or other cancer cell-related analytical methodologies. Another newly designed microfluidic technology called the JETTA ™ microfluidic chip, uses a size-and deformability-based capture scheme to characterize CTCs (Riahi *et al*, 2014). The novelty of this platform is that it segregates CTCs into their own chambers enabling single cell analysis based on morphological, immunologic, or genetic criteria. The spiral biochip uses inherent centrifugal forces for continuous, size-based, and EpCAM-independent separation of tumor cells. This device can process 3 ml of whole blood within an hour and has low leukocyte contamination (441 ± 319.5 leukocytes/ml) (Hou *et al*, 2013).

In addition to the exploitation of specific physical properties, alternative approaches can be used to enrich CTCs. The CAM (cell adhesion matrix) methodology (Vita Assay) enriches invasive and viable CTCs. Patient-derived blood samples are added to CAM-coated tubes at which invasive tumor cells are captured based on their preferential adhesion to the CAM. CTCs attach to CAM proteins and digest them by exploiting the mechanism by which metastatic tumor cells invade into surrounding tissues. Using this technique, CTCs (mean 61 CTCs/ml) were already found in 28 of 54 (52%) stage I-III breast cancer patients (Lu *et al*, 2010).

In summary, isolation of CTCs can be attained with various different strategies. More and more approaches for the enrichment appear on the market or are under development in laboratories all over the world. However, independent and large clinical studies are now required to determine the robustness and clinical validity of these new assays beyond the initial proof of concept.

### Identification and characterization of CTCs

Identification, enumeration, and molecular characterization of CTCs are likely to provide important insights into disease progression and might allow adaptation of therapeutic strategies. Differential CTC detection assays apply (or can be combined with) various strategies for the identification of tumor cells. Morphologic investigation together with fluorescence immunocytochemistry (ICC) is a common procedure for the identification and enumeration of CTCs after enrichment. The CellSearch ® system classifies a CTC as positive event if the cell is ≥ 4 μm, DAPI^+^ (4,6-diamino-2-phenylindole), pan-keratin^+^, and CD45^−^. The additional 4th fluorescence channel is accessible for a user-defined detection of, for example, therapy-relevant markers such as the androgen receptor (AR), the prostate-specific antigen (PSA), the human epidermal growth factor receptor 2 (ERBB2), the epidermal growth factor receptor (EGFR) (Riethdorf *et al*, 2007; Gasch *et al*, 2013), or EMT-associated molecules (such as N-cadherin or vimentin) (Armstrong *et al*, 2011). Identification of CTCs by ICC is also applicable when using other enrichment strategies (microfluidic or size-based platforms, the Gilupi CellCollector ™, red blood cell lysis, or density centrifugation-based procedures) (Nagrath *et al*, 2007; Maheswaran *et al*, 2008; Saucedo-Zeni *et al*, 2012; Babayan *et al*, 2013). However, conventional microscopic examination by a researcher is cost-and time-consuming. The Ariol ® system (Genetix USA) might be used for automated cell image capturing and analysis if the enriched cells are transferrable onto glass slides (Deng *et al*, 2008). Investigator microscopic filters set the limits for CTC enumeration and characterization by ICC; typically, the four different fluorescence channels are used for CTC characterization (two markers of interest), CD45 staining (to exclude false-positive findings), and nuclear counterstaining (DAPI or Hoechst33342).

Identification of CTCs by multiplex PCR targeting the numerous tumor-associated mRNA transcripts overcomes filter set limitations. CTC enrichment combined with a RT–PCR technology could already be used for the identification of tumor-related markers (*EpCAM*,*MUC1*, and *ERBB2*), EMT-associated transcripts (*PI3K*α (*phosphatidylinositol 3-kinase alpha*), *Akt-2*, or *Twist1*), or stem cell markers such as *ALDH1* (*aldehyde dehydrogenase 1*) (Kasimir-Bauer *et al*, 2012). Additional transcripts such as *KRT-19*,*MAGE-A3*, and *PBGD* have also been used for the detection and molecular characterization of circulating tumor cells (Strati *et al*, 2013). Hence, quantitative RT–PCR confers high sensitivity to CTC identification, although uncertainties are foreseeable with respect to specific primer and target gene selection. Additionally, accurate quantification of CTCs is difficult because CTCs are heterogeneous and may express different levels of the genes considered for identification. Furthermore, precise cut-off levels between mRNA amounts of normal blood cells and tumor cells must be determined and validated since tumor-associated targets such as *KRT-19* mRNA have also been found in a small number of healthy individuals (Stathopoulou *et al*, 2003). Hence, a combination of different genes should be used to improve the specificity of RT–PCR for reliable CTC detection.

Another strategy for the identification of CTCs is to enumerate and analyze proteins specifically secreted by viable tumor cells (EPISPOT, EPithelial Immuno SPOT). CTCs are enriched by negative depletion and subsequently cultured on a membrane coated with antibodies that capture the secreted proteins. Afterward, the proteins are readily identifiable by immunofluorescence microscopy using fluorochrome-labeled secondary antibodies targeting the protein of interest. Using this strategy, viable CTCs and DTCs have been already identified by the detection of secreted KRT-19, MUC1, PSA, or FGF-2 (Alix-Panabieres *et al*, 2007; Alix-Panabieres, 2012).

Taken together, different strategies can be used for CTC identification and characterization. Frequently, protein-(ICC), RNA-, and/or DNA-based (PCR) approaches are implemented. Each strategy has advantages and drawbacks. A combination of different analytical methodologies is likely to be beneficial and may encompass the heterogeneity of CTCs and help to understand their role in metastasis formation.

## Clinical implications of circulating tumor cells

DTCs in the bone marrow have been detected for all solid tumor types, suggesting that this compartment might be a preferred reservoir for blood-borne DTC from where they may re-disseminate to other distant organs such as liver or lungs where better growth conditions may exist. For example, DTCs are prognostic markers in colorectal cancer even though bone metastases are very rare in this cancer type (Lindemann *et al*, 1992; Pantel *et al*, 1993). In breast cancer, where bone metastases are more frequent, DTCs in the bone marrow may predict metastatic relapse in other organs as well (Braun *et al*, 2000, 2005). However, it cannot be excluded that DTCs may home equally well to other organs such as lung or liver, where however they may not be so easily detected as in bone marrow. The observed correlation between DTCs in bone marrow and local relapse in breast cancer (Bidard *et al*, 2008) is consistent with the recent experimental findings in mice that these cells might even re-infiltrate their primary organs and promote tumor progression (Kim *et al*, 2009). If the bone marrow is a preferred reservoir for DTCs, drugs targeting the BM–tumor interactions (e.g., bisphosphonates or antibodies to the RANK ligand (Lewiecki, 2011; Gronich & Rennert, 2013)) might help to prevent metastatic or even local relapse.

Sequential peripheral blood drawings in particular for real-time monitoring of minimal residual disease in cancer patients undergoing systemic therapies are clearly more acceptable than repeated bone marrow aspirations. Indeed, many research groups are currently assessing the clinical value of CTC analyses, which so far has been proven to provide significant prognostic information in metastatic breast cancer (Zhang *et al*, 2012; Bidard *et al*, 2014), and other solid tumors such as of the prostate (de Bono *et al*, 2008), colorectal (Cohen *et al*, 2008), and lung (Krebs *et al*, 2011; Hou *et al*, 2012) cancer, and seems to be superior over conventional imaging methods for response evaluation (Budd *et al*, 2006). However, the real challenge of CTC technologies is to monitor minimal residual disease in patients without signs of overt metastasis, as the CTC counts are very low in these patients. Promising results indicating that even such low CTC counts may have prognostic relevance have been recently published for several tumor entities such as breast cancer (Lucci *et al*, 2012; Rack *et al*, 2014), bladder cancer (Rink *et al*, 2011; Gazzaniga *et al*, 2014), and colorectal cancer (Deneve *et al*, 2013). Nevertheless, more sensitive methods and/or the analysis of larger amounts of blood (Saucedo-Zeni *et al*, 2012; Fischer *et al*, 2013) might be required to increase the robustness of CTC measurements in these patients. The implementation of leukapheresis or other innovative approaches for CTC enrichment (such as the CellCollector ™) enables the screening for CTCs in blood volumes of 1.5-25 liters. The analysis of higher blood volumes may significantly improve the capturing rates of CTCs, which might help to predict therapy response and monitoring of the disease especially in early-stage disease.

Furthermore, molecular characterization of CTCs might be essential to identify therapeutic targets and contribute to more ‘tailored’ and personalized anti-metastatic therapies. In current clinical practice, the decision on targeted therapies is solely based on the analysis of the primary tumor although the therapy is directed against metastatic cells (Wan *et al*, 2013). However, metastatic relapse may occur many years after primary tumor diagnosis and surgical resection (Uhr & Pantel, 2011). Thus, selection pressure during the complex metastatic process might have originated a particular subclone of cancer cells that is underrepresented in the primary tumor and may therefore be easily missed using next-generation sequencing of single CTCs (Heitzer *et al*, 2013; Lohr *et al*, 2014). In particular, the microenvironment at the distant metastatic site can alter expression patterns as compared to their ascendants in the primary tumor (Korkaya & Wicha, 2013). All considered, the analysis of metastatic cells should provide additional information not otherwise obtainable from the primary tumor. However, biopsies of metastases are invasive procedures and certain locations (e.g., lung or brain) are especially difficult to access. Thus, the analysis of CTCs in the peripheral blood, a ‘liquid biopsy’, might become a much less invasive and cost-effective alternative (Alix-Panabieres & Pantel, 2013). Metastatic cells located at different organs may replenish the pool of CTCs and their analysis may provide useful information on actual targets and resistance mechanisms of systemic anti-cancer therapies (Wan *et al*, 2013), which may in turn lead to a better selection of cancer patients for targeted therapies. In breast cancer, ERBB2 is one of the most prominent targets for systemic therapy (Wan *et al*, 2013). Currently, all patients are stratified to trastuzumab (or other anti-ERBB2 therapies) by primary tumor tissue analysis only. Recent reports, however, have shown that ERBB2-positive CTCs can be detected also in patients with ERBB2-negative primary tumors (Riethdorf *et al*, 2010; Ignatiadis *et al*, 2011; Hartkopf *et al*, 2012), suggesting that there might exist additional patients that could benefit from ERBB2-directed therapies. Ongoing clinical studies (e.g., DETECT III (NCT01619111) trial in Germany and CTC-TREAT (NCT01548677) trial in Europe) will reveal whether the ERBB2 status of CTCs may predict response to ERBB2-directed therapies (Bidard *et al*, 2013a,b).

Another example for the use of CTCs as predictive biomarkers is the presence of ER-negative CTCs in breast cancer patients with ER-positive primary tumors (Babayan *et al*, 2013). ER is the most common therapeutic target in breast cancer, and 70–80% of patients have ER-positive primary tumors. However, ER-negative CTCs that may have escaped hormonal therapy to block ER-mediated growth frequently occur in these patients.

Genomic CTC analysis can also reveal gene mutations relevant for therapy resistance. For example, *KRAS* mutations are known to block the effect of therapeutic EGFR inhibition by antibodies or small inhibitors in colorectal cancer patients (Wan *et al*, 2013). The analysis of individual CTCs has shown a remarkable intra-patient *KRAS* mutation heterogeneity (i.e., *KRAS*^*WT*^ and *KRAS*^*MT*^ CTCs are present in the same patient) (Gasch *et al*, 2013). Indeed, the presence of *KRAS*-mutated CTCs in patients with *KRAS* wild-type primary colon carcinomas might be one explanation for failure of drug-mediated EGFR inhibition in these patients (Douillard *et al*, 2013; Peeters *et al*, 2013). At present, however, only an arbitrary section of the primary tumor is analyzed for *KRAS* mutations in colorectal cancer patients and the genomic heterogeneity of metastatic cells—the actual targets of systemic therapy–is not taken into consideration for therapy decisions.

In conclusion, the characterization of CTCs may have an important impact as companion diagnostics in future clinical trials testing new targeted therapies (Wan *et al*, 2013).

## Conclusions and outlook

Metastasis starts with cancer cell invasion from the primary tumor through the surrounding tissue into the bloodstream. Tumor cell motility may be acquired by different processes of which epithelial-to-mesenchymal transition (EMT) and amplification of centrosomes are currently considered to be the main ones. EMT leads to (partial) loss of the epithelial characteristics of cancer cells, whereas centrosome amplification-associated spread may retain the expression of epithelial markers. Because of these different dissemination processes, what the true features of the metastasis-initiating cells are and whether these cells can be found among the CTCs that can currently be detected in the blood of cancer patients remain to be fully established. CTC enrichment methods can broadly be divided into EpCAM-dependent cell selection that allows the identification of epithelial tumor cells and on the other hand the EpCAM-independent cell selection methods that may help to identify additional mesenchymal-like CTC subpopulations. Interestingly, the first report on grafting human-derived breast cancer CTCs in xenograft assays indicated that the putative metastasis-initiating cells expressed EpCAM, although at a reduced level (Baccelli *et al*, 2013). CTC detection approaches are usually based on the expression or secretion of specific proteins, DNA mutations, or RNA profiling. The development of new enrichment technologies are currently aimed at increased sensitivity and investigating increased blood volume using ‘*in vivo* strategies’ to gain purity and enumeration of higher CTC counts.

Clinically, quantification of CTCs is of high value as these cancer cells generally represent the tumor (metastases) and facilitate real-time monitoring during systemic therapies by sequential peripheral blood sampling. Furthermore, molecular characterization of CTCs might enable the identification of therapeutic targets and contribute to personalized anti-metastatic therapies. Proof of the clinical relevance of the detection and characterization of CTCs has been substantially accumulating during the past decades. The use of xenograft models is a promising approach to gain further insights into the biology of tumor cell dissemination and may further help to test responses to newly designed therapies (Baccelli *et al*, 2013; Hodgkinson *et al*, 2014; Yu *et al*, 2014).

In conclusion, analysis of CTCs in the peripheral blood (liquid biopsy) has a clear potential to further our understanding of the biology of tumor cell dissemination and to improve the management and possibly the prevention of metastatic disease in the near future.

## Conflict of interest

The authors declare that they have no conflict of interest.

**Pending issues**

Do the currently used CTC enrichment and detection techniques allow us to identify bona fide metastasis-initiating cells (MICs)?

Are EMT and MET required for tumor cell dissemination and metastasis outgrowth or are non-EMT events more effective in causing metastatic dissemination?

Can CTCs be used to investigate the effectiveness of cancer treatment and are CTCs furthermore reliable targets to predict personalized treatment strategies based on a blood test (‘liquid biopsy’)?

## Abbreviations

Cadherins: Calcium-dependent cell adhesion proteins involved in mechanisms regulating cell–cell adhesion, mobility, and proliferation of epithelial cells.
Centromere: A condensed and constricted region of a chromosome, to which the spindle fiber is attached during mitosis.
Claudins: Important components of the tight junctions. Claudins are transmembrane proteins and establish the paracellular barrier, which controls the flow of molecules in the intercellular space between the cells of an epithelium.
Circulating tumor cell (CTC): Cell that detached into the vasculature from a primary tumor or metastasis and can be found in the bloodstream of cancer patients.
Disseminating tumor cell (DTC): Settlement of CTCs in secondary organs, such as liver, bone, and lungs. DTCs may stay in a dormant state or giving rise to an overt metastasis.
Epithelial cell adhesion molecule (EpCAM): Cell adhesion molecule that is overexpressed in many carcinomas and commonly used as a marker for CTC enrichment.
Epithelial mesenchymal transition (EMT): A biological process by which epithelial cells lose their cell polarity and cell–cell adhesion and gain migratory and invasive properties, thus acquiring a mesenchymal-like cell phenotype.
Mobile cancer cell: A cell that moves due to external forces.
Motile cancer cell: A cell that is able to move on its own accord.
Tumor cell plasticity: The ability of a tumor cell to adapt to a new microenvironment and reverse the adaptation if needed
